# Oxytocin Receptor Expression and Activation in Parasympathetic Brainstem Cardiac Vagal Neurons

**DOI:** 10.1523/ENEURO.0204-25.2025

**Published:** 2025-08-13

**Authors:** Xin Wang, Caitlin Ribeiro, Anna Nilsson, Joan B. Escobar, Bridget R. Alber, John R. Bethea, Vsevolod Y. Polotsky, Matthew W. Kay, Kathryn Schunke, David Mendelowitz

**Affiliations:** ^1^Department of Pharmacology and Physiology, The George Washington University, Washington, DC 20037; ^2^Department of Cell and Molecular Biology, University of Hawaii, Honolulu, Hawaii 96813; ^3^Department of Biomedical Engineering, The George Washington University, Science & Engineering Hall, Washington, DC 20052; ^4^Departments of Anatomy and Cell Biology, George Washington University, Washington, DC 20037; ^5^Anesthesiology and Critical Care Medicine, George Washington University, Washington, DC 20037

**Keywords:** autonomic regulation, cardiac vagal neurons, chemogenetic, DMNX and NA, DREADDs, oxytocin receptors

## Abstract

Autonomic imbalance—particularly reduced activity from brainstem parasympathetic cardiac vagal neurons (CVNs)—is a major characteristic of many cardiorespiratory diseases. Therapeutic approaches to selectively enhance CVN activity have been limited by the lack of defined, translationally relevant targets. Previous studies have identified an important excitatory synaptic pathway from oxytocin (OXT) neurons in the paraventricular nucleus of the hypothalamus to brainstem CVNs, suggesting that OXT could provide a key selective excitation of CVNs. In clinical studies, intranasal OXT has been shown to increase parasympathetic cardiac activity, improve autonomic balance, and reduce obstructive event durations and oxygen desaturations in obstructive sleep apnea patients. However, the mechanisms by which activation of hypothalamic OXT neurons, or intranasal OXT, enhance brainstem parasympathetic cardiac activity remain unclear. CVNs are located in two cholinergic brainstem nuclei: nucleus ambiguus (NA) and dorsal motor nucleus of the vagus (DMNX). In this study, we characterize the colocalization of OXT receptors (OXTRs) in both CVNs and non-CVN cholinergic neurons in the male and female mouse NA and DMNX nuclei. We found that OXT receptors are highly expressed in CVNs in the DMNX, but not in the NA. OXT increases the firing of DMNX CVN, with no effect on NA CVNs. Selective chemogenetic excitation of OXTR+ CVNs in the DMNX—achieved by a combination of Cre- and flp-dependent DREADD expression—evoked a rapid and sustained bradycardia. These findings suggest that activation of DMNX CVNs expressing OXTR with oxytocin may represent a novel translational therapeutic target for restoring autonomic balance in cardiorespiratory disorders.

## Significance Statement

In this study, we characterize the colocalization of oxytocin receptors (OXTRs) in cardiac vagal neurons (CVNs), as well as non-CVN cholinergic neurons, located in the nucleus ambiguus (NA) and dorsal motor nucleus of the vagus (DMNX) nuclei. We found that OXTRs are highly expressed in CVNs in the DMNX, but not in those within the NA. OXT increased the firing rate of CVNs in the DMNX, while having no effect on CVNs in the NA. Selective chemogenetic excitation of OXTR-positive CVNs in the DMNX evoked a rapid and sustained bradycardia. These findings suggest that activation of DMNX CVNs via OXT signaling could represent a novel translational therapeutic strategy to enhance autonomic balance in cardiorespiratory diseases.

## Introduction

Autonomic imbalance, particularly reduced activity from brainstem parasympathetic cardiac vagal neurons (CVNs), is a major characteristic of many cardiorespiratory diseases including hypertension, sudden cardiac death, sleep apnea, heart failure, sudden unexpected death in epilepsy, as well as other diseases such as obesity, anxiety, and osteoarthritis (Jones et al., 1998; Cheshire, 2013; Kay et al., 2022; Strain et al., 2023, 2024; Sohn et al., 2024). Therapeutic approaches to selectively increase parasympathetic cardiac activity have been limited by lack of identified translational targets in CVNs. Previous work has shown that there is an important excitatory synaptic pathway from oxytocin (OXT) neurons in the paraventricular nucleus of the hypothalamus (PVN) to brainstem CVNs and that this pathway provides a key excitation to CVNs that are inherently silent (Mendelowitz, 1996; Pinol et al., 2012). Selective chemogenetic activation of PVN OXT neurons, using Designer Receptors Exclusively Activated by Designer Drugs (DREADDs), reduces heart rate (HR) and blood pressure by increasing cardiac vagal tone (Dyavanapalli et al., 2020b). Activation of PVN OXT neurons, as well as intranasal OXT, increases parasympathetic activity to the heart, restores autonomic balance, and prevents or mitigates the adverse cardiorespiratory dysregulation that occurs in many diseases with autonomic imbalance, including chronic intermittent hypoxia induced hypertension (Rodriguez et al., 2023), heart failure (Garrott et al., 2017; Dyavanapalli et al., 2020a,b), and myocardial infarction (Schunke et al., 2023). In clinical studies, intranasal OXT increases parasympathetic cardiac activity, improves autonomic balance, and reduces obstructive event durations and oxygen desaturations in obstructive sleep apnea patients (Jain et al., 2017, 2020).

However, the mechanisms by which activation of hypothalamic OXT neurons, or intranasal OXT, increases brainstem parasympathetic cardiac activity are poorly understood. CVNs are located in two cholinergic brainstem nuclei: the nucleus ambiguous (NA) and dorsal motor nucleus of the vagus (DMNX). In this study we characterize the colocalization of OXT receptors (OXTRs) in CVNs, as well as non-CVN cholinergic neurons, located in the NA and DMNX nuclei. Selective chemogenetic excitation of OXTR+ CVNs was performed by expressing DREADDs with a combination of Cre- and flp-dependent viruses. We found that OXT receptors are highly expressed in CVNs in the DMNX and OXT increases DMNX CVN activity, but the receptors and responses to OXT are absent in CVNs in the NA. Selective chemogenetic activation of OXTR+ CVNs in the DMNX evoked a rapid and sustained bradycardia. Activation of DMNX CVNs with OXT could be a novel translational therapeutic target to increase autonomic balance in cardiorespiratory diseases.

## Materials and Methods

### Animals

All animal experiments were performed in accordance with NIH and Institutional Animal Care and Use Guidelines, approved by the George Washington University Institutional Animal Care and Use Committee (IACUC, protocol #2022-028). The transgenic OXTR-Cre mouse line (B6.Cg-*Oxtr^tm1.1(cre)Hze^*/J, Common Name: Oxtr-T2A-Cre-D, Jackson Laboratory, stock #031303) and the Cre-dependent floxed ChR2-eGFP mouse line (ROSA26-EGFP Cre recombinase report mouse, Jackson Laboratory, stock #012569) were used in the study. Mice were housed at standard environmental conditions (24–26°C in the 12 h light/dark cycle, 7 A.M.–7 P.M. lights on), until the experiments started. Water and food were available *ad libitum*.

Mice were crossbred to express ChR2-eYFP in OXTR+ neurons. One parent (OXTR-Cre mouse) had homozygous expression of an OXTR promoter to guide the expression of Cre recombinase while the other parent (Cre-dependent floxed ChR2-eYFP mouse) had homozygous ChR2-eYFP fusion protein expression dependent upon Cre expression. Offspring (OXTR-ChR2-EYFP) were genotyped by Transnetyx before experiments. A total of 25 mice (11 males and 14 females) have been used in the study.

### Retrograde labeling of preganglionic parasympathetic CVNs

To label CVNs in the NA and DMNX, cholera toxin B (CTB) conjugated to Alexa Fluor 555 (Invitrogen, C22843) was injected into the right atrial pericardial space of postnatal day 5 (P5) OXTR-ChR2-EYFP mice. Following injection, mice recovered on a 37°C heating pad until regaining the righting reflex, after which they were returned to the litter. Three to seven days postinjection, CVNs in the brainstem NA and DMNX nuclei were identified by the presence of fluorescent tracer (red; CTB-Alexa Fluor 555). Eight animals (three males and five females) from three pairs of crossbred mice were used for this study.

### Selective DREADD expression in OXTR+ CVNs

To selectively express DREADDs in DMNX OXTR+ CVNs, we used a two-stage combination of Cre- and FlpO-expressing viruses in a separate group of mice (*n* = 7, four males and three females). A retrograde Cre-dependent site-specific recombinase FlpO virus (AAVrg pEF1a-DIO-FLPo-WPRE-hGHpA, Addgene #87306; 1 µl, ≥7 × 10^12^ vg/ml) was injected into the pericardial sac of at P5 OXTR-ChR2-EYFP mice (same procedure as above) in an initial surgery. Four weeks later, the Flp-dependent excitatory hM3D (Gq) DREADD mCherry virus [pAAV-hSyn-fDIO-hM3D(Gq)-mCherry-WPREpA (AAV8), ≥1 × 10^13^ vg/ml, Addgene viral prep #154868-AAV8] was injected into the DMNX. Briefly, mice were anesthetized using a mixture of ketamine (100 mg/kg) and xylazine (10 mg/kg) and then secured in a stereotaxic frame (David Kopf Instruments). Using stereotaxic guidance of a pulled glass capillary (40 µm tip diameter; G1, Narishige) attached to a pneumatic microinjector (IM-11-2, Narishige), 240 nl of viral preparation was bilaterally injected into the DMNX (AP 0, at the level of obex, ML 0.15 mm, DV 0.5 mm). The capillary remained in place for 5 min to allow diffusion and was then removed slowly to avoid dispersion to neighboring brainstem regions. DREADDs expressing DMNX parasympathetic CVNs were identified by the presence of mCherry.

### Brain slice preparation, immunohistochemistry, and RNAscope in situ hybridization

One week after injecting Alexa Fluor 555-conjugated cholera toxin subunit B into the pericardial sac, P12–P14 pups were anesthetized by isoflurane and transcardially perfused with phosphate-buffered saline (PBS) followed by 4% paraformaldehyde (PFA). Brains were dissected, postfixed in 4% PFA overnight at room temperature, and then washed three times for 10 min in PBS. A serial of 50 µm coronal section medullary slices containing the NA and/or DMNX were obtained using a Leica dissection vibratome (Leica VT 1000S). A total of 10–20 NA and 9–11 DMNX medullary slices were obtained from each animal. All slice sections were processed using immunohistochemistry for choline acetyltransferase (ChAT) and eYFP to identify cholinergic and OXTR-expressing neurons, respectively. After blocking nonspecific proteins in 10% normal goat serum (NGS) in PBS with 0.3% Triton X-100 (PBST) for 4 h at room temperature, slices were incubated in primary antibody at 4°C for 24 h (mouse monoclonal-ChAT, diluted 1:500, Thermo Fisher Scientific, catalog #MA5-31382; and chicken anti-GFP/EYFP, 1:1,500 dilution; Abcam, ab 13970). Secondary antibodies were applied for 4 h at room temperature. Secondary antibodies were goat anti-mouse antibody conjugated with Alexa Fluor 647 (Thermo Fisher Scientific, catalog #A-21241) and goat anti chicken Alexa Fluor 488 (Thermo Fisher Scientific, catalog #A 11039), both 1:200 dilution. Slides were mounted with Fluoro-Gel (Electron Microscopy Sciences, SKU: 17985-10) and imaged with a Leica TCS SP8 multiphoton confocal microscope equipped with supercontinuum white laser source and single molecule detection hybrid detectors (SMD HyD, Leica).

For RNAscope in situ hybridization (ISH) experiments, three animals (one male and two females) were anesthetized by isoflurane and transcardially perfused with RNAse-free PBS, followed by 4% PFA. Brain tissues were obtained and fixed in 4% PFA solution for 4 h at 4°C. After fixation, brains were transferred into RNAse-free 30% sucrose (PBS, for dehydration and cryoprotection) and stored at 4°C for 48 h, then stored at −80°C, and cryosectioned coronally 12 µm thick. Every fourth section was prepared according to the manufacturer's instructions, using the RNAscope Multiplex Fluorescent Detection Reagents v2 kit (ACDBio, 323110) combined with Integrated Co-Detection (ACDBio, 323180). Briefly, slides were baked at 60°C, fixed in 4% PFA, and dehydrated. Slides were incubated in Hydrogen Peroxide (ACDBio, 322335), followed by antigen retrieval using RNAscope 1× Co-Detection Target Retrieval buffer (ACDBio 323165) heated to 99°C. Primary antibody to detect OXTR-containing neurons (Chicken anti-GFP/EYPF; Abcam, AB13970, 1:1,500) was incubated overnight at 4°C. Slides were postfixed using 10% NMBF followed by the RNAscope Multiplex Fluorescent Detection protocol according to the manufacturer's instructions. RNAscope Probe C1 mmOxtr (ACDBio, 412171) was used to target OXT receptor mRNA, and Vivid Fluorophore 650 (ACDBio, 323273, 1:500) was used to label the OXTR probe. Finally, slides were incubated in secondary antibody Alexa Fluor Goat anti-chicken 488 (Invitrogen, A11039, 1:200), stained with DAPI (ACDBio, 323108), mounted with ProLong Gold Antifade Mountant (Invitrogen, P36934), and stored at 4°C until imaging.

### Patch-clamp electrophysiology experiments

To study the effect of OXT on CVN activity, OXTR-ChR2-EGFP mice (three males and four females) received CTB pericardial sac injections at P5, and whole-cell patch-clamp recordings of CVNs occurred at P8–P12. Animals were anesthetized with isoflurane, killed, and transcardially perfused with ice-cold NMDG aCSF as follows (in mM): 93 NMDG, 93 HCl, 2.5 KCl, 1.2 NaH_2_PO_4_, 30 NaHCO_3_, 25 glucose, 20 HEPES, 5 sodium ascorbate, 2 thiourea, 3 sodium pyruvate, 10 MgSO_4_.7H_2_O, 0.5 CaCl_2_.2H_2_O, oxygenated with 95% O_2_ and 5% CO_2_ (pH 7.4). The brain was carefully removed, and brainstem slices (250 µm) were prepared using a vibratome. Tissue slices were then moved from the bath solution and incubated in the NMDG recovery solution as follows (in mM): 93 NMDG, 93 HCl, 2.5 KCl, 1.2 NaH_2_PO_4_, 25 NaHCO_3_, 20 HEPES, 25 d-glucose, 10 MgSO_4_, 0.5 CaCl_2_ bubbled with 95% O_2_/5% CO_2_ at 34°C for 10 min. Tissue slices were then moved to a recording chamber and perfused with standard aCSF solution containing the following (in mM): 125 NaCl, 3 KCl, 25 NaHCO_3_, 5 HEPES, 5 d-glucose, 1 MgSO_4_, 2 CaCl_2_ and continuously bubbled with 95% O_2_/5% CO_2_ to maintain pH at 7.4 at room temperature (22–24°C). Patch electrodes were filled with an intracellular recording solution at pH 7.3 containing the following (in mM): 130 K-gluconate, 4 KCl, 10 HEPES, 0.3 EGTA, 4 Mg-ATP, 0.3 Na_2_-GTP, 10 phosphocreatine-Na_2_, and 13.4 biocytin. Biocytin [0.5%, a highly photostable far-red (excitation laser 640 nm) biocytin fluorophore conjugated dye, CF640R, Biotium] was added in the patch solution to further identify the neurons in the NA and DMNX using immunohistochemistry staining. Identified CVNs were voltage clamped at a holding potential of −80 mV. Gabazine (25 µM) was used to block GABAergic inhibitory neurotransmission, and strychnine (1 µM) was included to block glycinergic inhibitory neurotransmission. Focal drug application was performed using a PV830 Pneumatic PicoPump pressure delivery system (World Precision Instruments). Drugs were ejected from a patch pipette positioned within 30 µm from the patched CVN. The maximum range of drug application has been previously determined to 100–120 µm downstream from the drug pipette and considerably less behind the drug pipette. Electrophysiological data were digitized and collected via Clampex (10.2) and analyzed using Clampfit (10.7).

### Confocal imaging

Leica TCS SP8 MP confocal microscopy was used to assess colocalization of ChR2-eGFP-labeled OXTR+ neurons, CVNs, and ChAT (cholinergic) neurons in the DMNX and NA. Tissue slices containing the DMNX and NA were examined with 418, 514, and 610 nm wavelengths to visualize eGFP, CTB-Alexa Fluor 555, and the ChAT-Alexa Fluor 647, respectively. Images were captured with a DFC365FX camera at 2,048 by 2,048 pixel resolution. Full-field images of the entire slice were taken at 10× to localize the NA and DMNX. *Z*-stacks were then taken with 20×/0.75 oil-immersion objective, at *z*-step size of 0.9 µm to produce image volumes allowing for the identification of colocalization of ChAT, CVNs, and ChR2-eGFP-positive neurons. Images were processed and analyzed using Imaris 10 software (Oxford Instruments).

Brain slices for RNAscope ISH were imaged with the automated Leica DMi8 Thunder microscope mounted with a Hamamatsu ORCA-Flash4.0 v3 camera and paired with advanced LAS X software for OXTR mRNA detection. A 63×/1.40 oil-immersion objective was used to image processed sections, illuminated with the LED8 spectrum to visualize GFP-eYFP-488, OXTR mRNA-Vivid 650, CTB-Alexa Fluor 555, and DAPI. A total of 6–10 sections spanning the DMNX from two mice were analyzed. Rostral sections of the NA in the brainstem showed no OXTR-positive cells and were not quantified further.

### In vivo heart rate measurements

One month after DREADD injection, animals were implanted with a telemetry device (DSI wireless transmitters, ETA-F10; Data Sciences International) with electrocardiographic (ECG) leads to measure HR. One week later, animals were acclimated to the whole-body plethysmography system (Scireq) for 3 h on 3 consecutive days. Control ECG and respiratory rate (RR) data were recorded following an intraperitoneal injection of either saline or clozapine-*N*-oxide (CNO; 1 mg/kg) to activate excitatory DREADDs in OXTR+ CVNs. HR data were obtained by analyzing the raw ECG signal using the ECG analysis platform of LabChart (ADInstruments). RR data were obtained using EMKA IOX Software and analyzed using the same approaches.

It is worth pointing out the differences in developmental timescales between in vitro and in vivo experiments. We use cholera toxin B (CTB) to retrogradely label CVNs in animals at age P5, conducting electrophysiology patch-clamp recordings and confocal imaging between P8 and P15. Our previous experiments have shown that CTB takes 24–48 h to reach the brainstem preganglionic parasympathetic CVNs in the NA and DMNX from the pericardial space injection site with peak fluoresce observed between 3 and 10 d post injection. This time window was chosen to ensure robust labeling while maximizing animal survive and recovery (>95%), which is significantly higher than in younger pups. In contrast, for in vivo plethysmography recording, we employed a dual viral strategy (Cre- and Flpo-dependent viral vectors) to selectively label and activate cardiac-projecting CVNs in the brainstem. This method requires ∼6 weeks for complete two-step viral expression. To address any potential developmental differences in OXTR expression, we perform the RNA scope experiments in adult animals, which confirmed OXTR mRNA expression in OXTR-Cre+ neurons in DMNX CVNs in adults.

### Data analysis

All data are presented as mean ± SEM. For ex vivo experiments, “*n*” is reported as number of recorded cells and the number of animals. For in vivo experiments, “*n*” is the number of animals. These values are stated throughout the Results and figure legends. Statistical comparisons were made using repeated-measures (RM) one-way and two-way ANOVA with Dunnett's and Tukey's multiple comparisons, paired or unpaired Student's tests, as appropriate. Specific statistical tests are noted in the results. Differences were considered statistically significant if *p* value <0.05. Graph creation and statistical analyses were conducted using GraphPad Prism 9 (GraphPad Software).

## Results

### Percentage of CVNs-ChAT neurons in the DMNX and NA in OXTR-Cre- ChR2-eYFP mice

**Figure 1.**
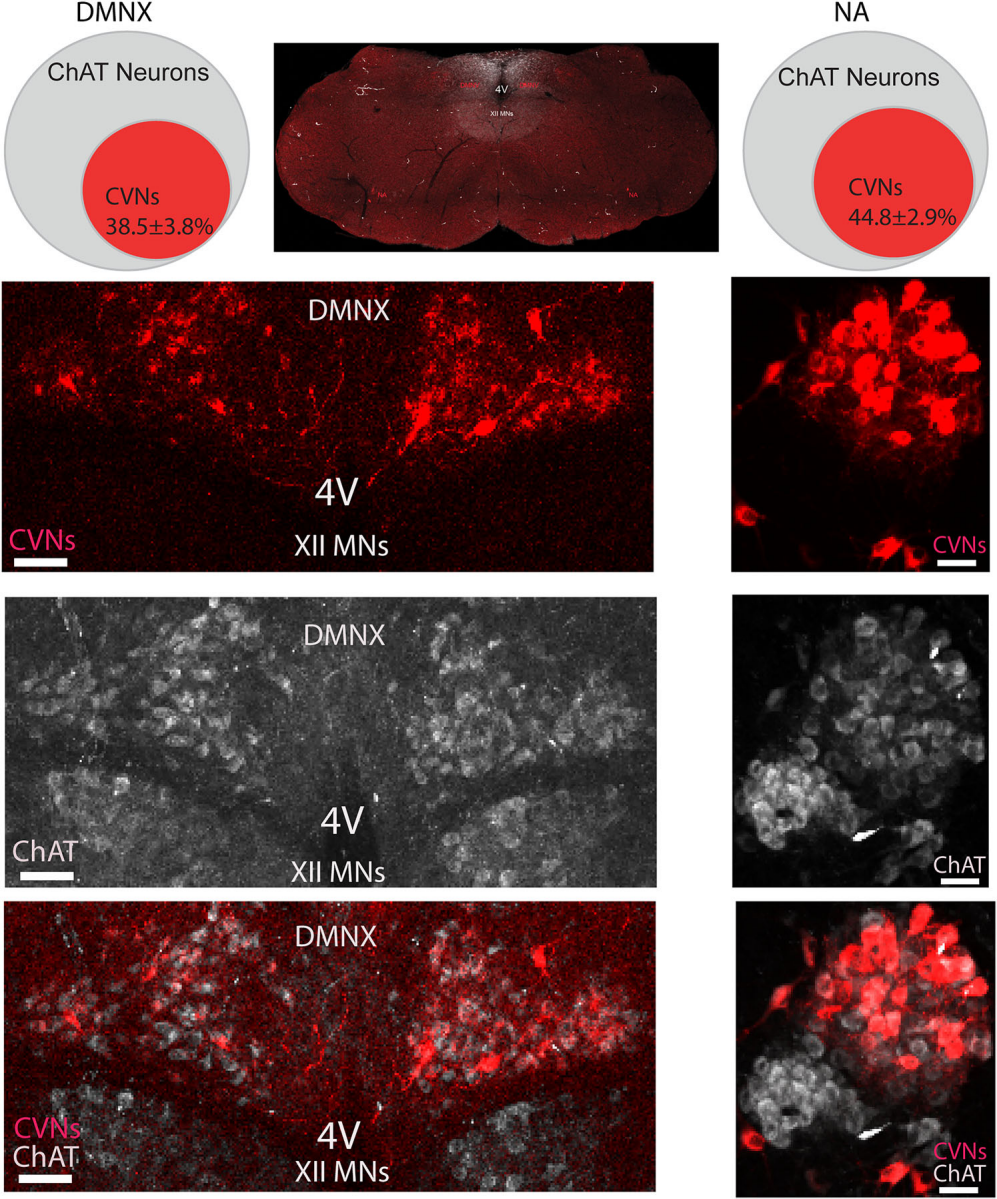
Percentage of CVNs-ChAT neurons in the DMNX and NA in OXTR-Cre- ChR2-eYFP male and female mice. Top middle, An example confocal image showing overview of brainstem containing CVNs (red) in the DMNX and NA. The DMNX data presented on the left and NA on the right panel. The top two Venn diagrams showing CVNs account for the percentage population of the cholinergic neurons in the DMNX (left) and NA (right). Example confocal images showing the CVNs in red (top), ChAT in gray (middle), and merged CVNs and ChAT neurons light red (bottom) in the DMNX and NA, respectively. Scale bar, 50 µm. For this and all the following figures: 4V, the 4th ventricle; XII MNs, hypoglossal motor neurons; CVNs, cardiac vagal neurons; ChAT, cholinergic neurons.

A detailed quantitative analysis of CTB-Alexa Fluor 555-labeled parasympathetic CVNs was conducted on brainstem tissue from animals which received pericardiac sac CTB-Alexa Fluor 555 injections. CVNs were nearly equally distributed in the NA and DMNX nuclei (467 ± 97 CVNs in the DMNX and 439 ± 53 CVNs in the NA per animal). As expected, CVNs in both nuclei were highly colocalized with ChAT (93 ± 1% in the DMNX and 95 ± 1% in the NA, *n* = 7 and *n* = 8 animals, respectively). CVNs comprised ∼40% of the ChAT population in these nuclei, with the population of CVNs consisting of 38.5 ± 3.8% of the ChAT neurons in the DMNX (*n* = 7 animals, 3 males and 4 females) and 44.8 ± 2.9% of the ChAT neurons in the NA (*n* = 8 animals, 3 males and 5 females; Fig.1).

### Colocalization of OXTR+ neurons with CVNs and non-CVN ChAT cells in the DMNX and NA

**Figure 2.**
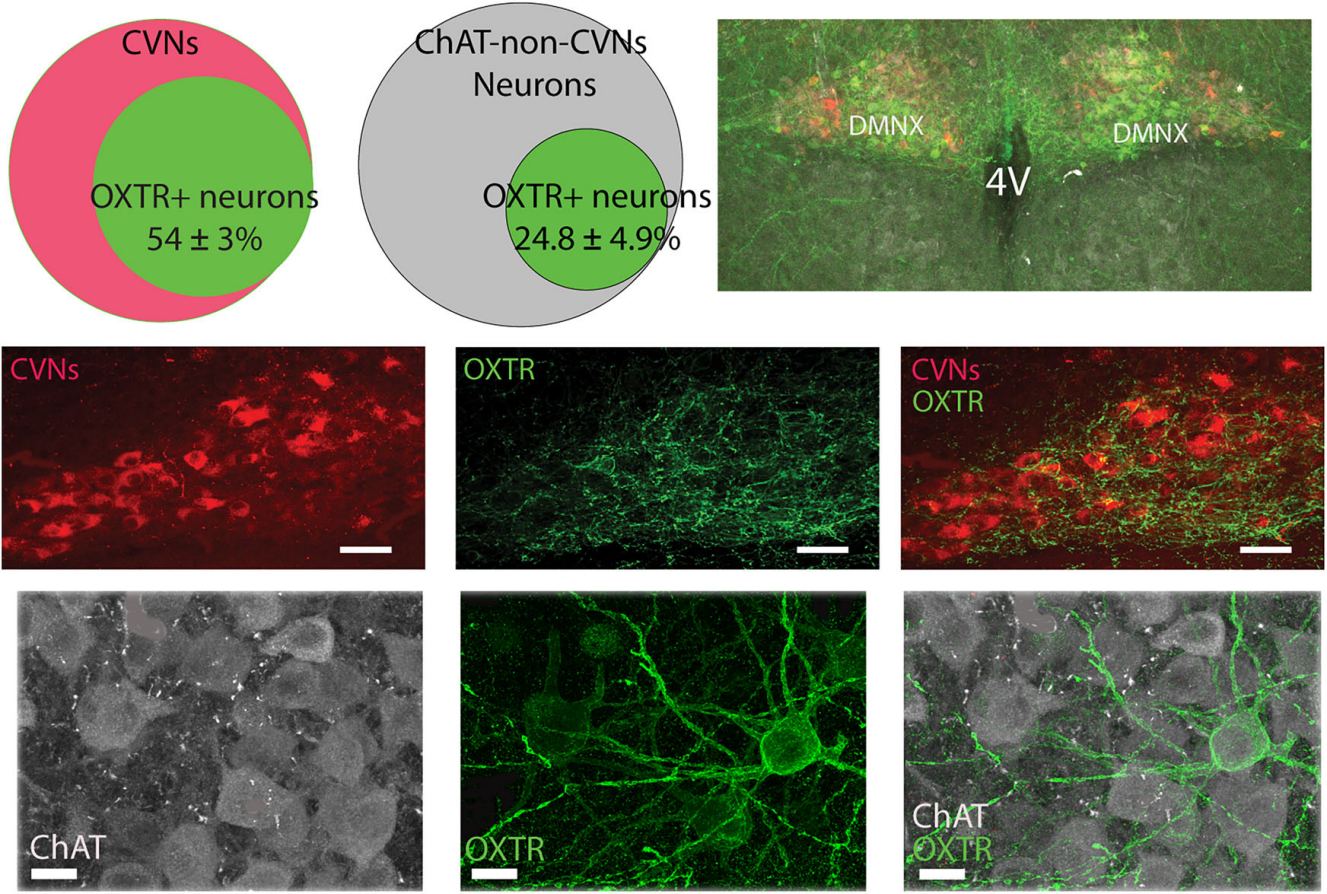
Colocalization of OXTR+ neurons with CVNs and non-CVN ChAT cells in the DMNX. Top right, An example of a low-resolution confocal image showing overview of brainstem dorsal side DMNX area containing CVNs (red), ChAT (gray), and OXTR+ (green). Top left two Venn diagrams showing the percentage of CVNs and non-CVN-ChAT neurons containing OXTR, respectively. The middle panel represents CVNs colocalization with OXTR+ neurons: the confocal images mark CVNs in red (left), OXTR+ in green (middle), and CVNs merged with OXTR+ labeling (right); scale bar, 20 µm. The bottom panel illustrates the OXTR+ neurons colocalized with ChAT neurons that are not CVNs. Typical confocal images illustrate ChAT neurons in gray (left), OXTR+ neurons in green (middle), and merged OXTR+ and ChAT neurons (right); scale bar, 10 µm. OXTR, oxytocin receptor.

**Figure 3.**
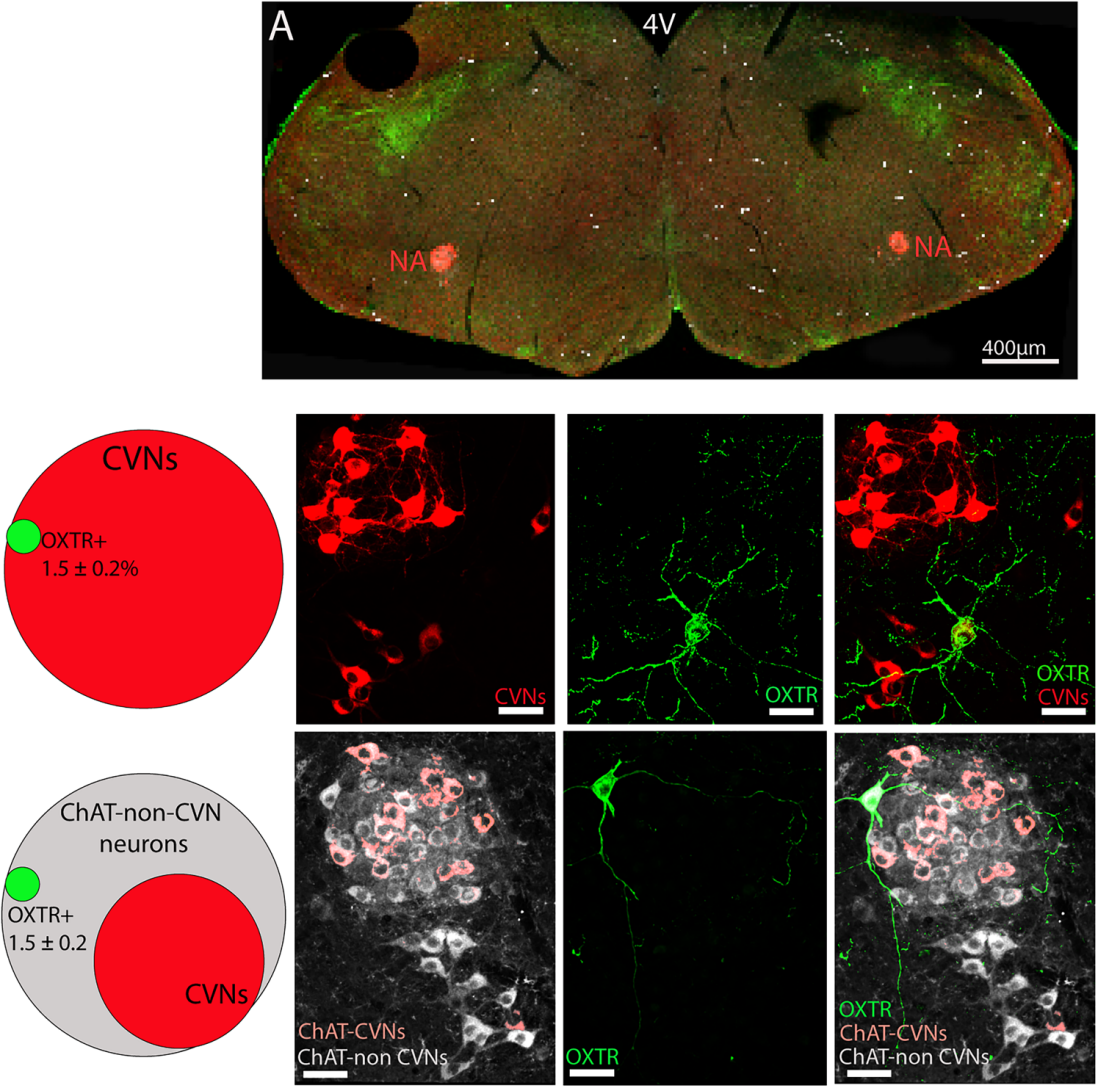
The OXTR+ neurons colocalization with CVNs and non-CVN ChAT cells in the NA. A: The low-resolution confocal image showing overview of a brainstem containing CVNs (red), ChAT (gray), and OXTR+ (green) in the NA. The middle panel reveals the percentage of CVNs containing OXTR+ (Venn diagram on the left), confocal images represent CVNs in red (2nd left), OXTR+ neurons in green (2nd from right), and colocalized OXTR+ neuron with CVNs (right). The bottom panel denotes the percentage of non-CVN ChAT neurons containing OXTR+ (Venn diagram on the left), confocal images showing ChAT in gray (2nd left), OXTR+ neurons in green (2nd from right), and colocalized OXTR+ neuron with non-CVN ChAT neurons (right). Scale bar, 50 µm.

OXTR+ neurons were highly prevalent within the DMNX (746 ± 199 OXTR+ neurons/animal, *n* = 7 animals, 3 males and 4 females), but there were sparse in the NA (74 ± 14 OXTR+ neurons/animal, *n* = 8 animals, 3 males and 5 females), as illustrated in Figure 2. A majority of CVNs in the DMNX were OXTR+ as 54 ± 3% of CVNs in the DMNX were also OXTR+; see Figure 2. Some non-CVN DMNX neurons also colocalized with OXTR+, 24.8 ± 4.9%. In contrast, very few CVNs in the NA were OXTR+ neurons (1.5 ± 0.2%) and similarly a small number of non-CVN ChAT neurons in the NA were OXTR+ neurons (1.5 ± 0.2%; Fig. 3).

### ChR2-eGFP-expressing neurons are correlated with active OXTR mRNA transcription

**Figure 4.**
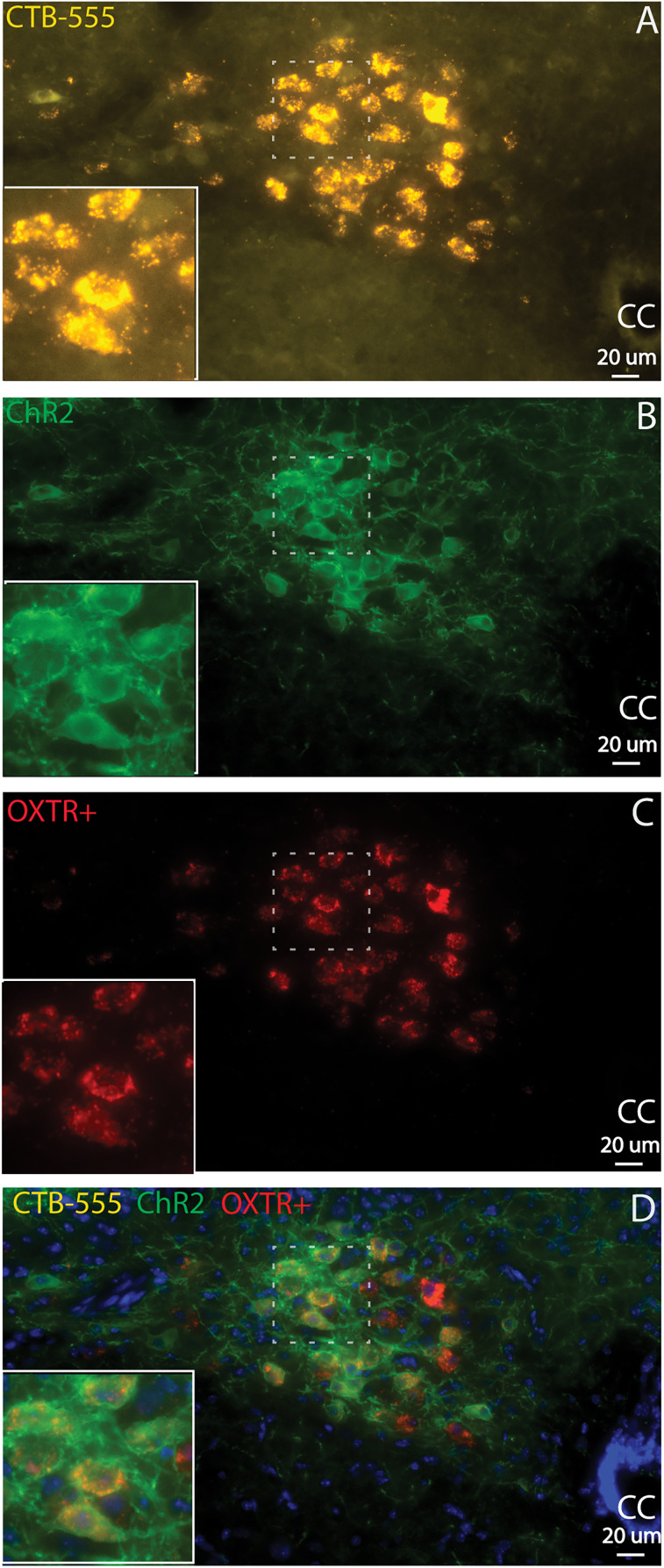
ChR2-eGFP-expressing neurons are correlated with active OXTR mRNA transcription in adult crossbred OXTR+ Cre × floxed ChR2-eGFP animals. ***A***, CTB-555 labeled CVNs (gold); ***B***, ChR2-eGFP-labeled OXTR^+^ neurons and fibers (green); ***C***, OXTR mRNA+ cells (red); and ***D***, all of OXTR^+^-Cre ChR2-positive neurons (green) colocalized with the OXTR mRNA probe (red). Insert, focus on the dash line marked area. cc, central canal.

To test whether the crossbred OXTR+ cre × floxed ChR2-eGFP male and female animals express OXTR mRNA as adults, we performed RNAscope ISH experiments on brain sections from three adult OXTR-Cre-ChR2-eGFP mice (one males and two females). As shown in Figure 4, eGFP-labeled OXTR+-cre ChR2-positive neurons (green) showed strong colocalization with the OXTR mRNA probe (red), with 98.6% of OXTR mRNA+ cells also expressing ChR2-eGFP. Furthermore, 100% of the OXTR-Cre-ChR2-eGFP-positive neurons were colocalized with OXTR mRNA. No OXTR mRNA+ neurons were found in the NA (data not shown).

### Changes in action potential firing rate in OXTR+ CVNs with OXT

**Figure 5.**
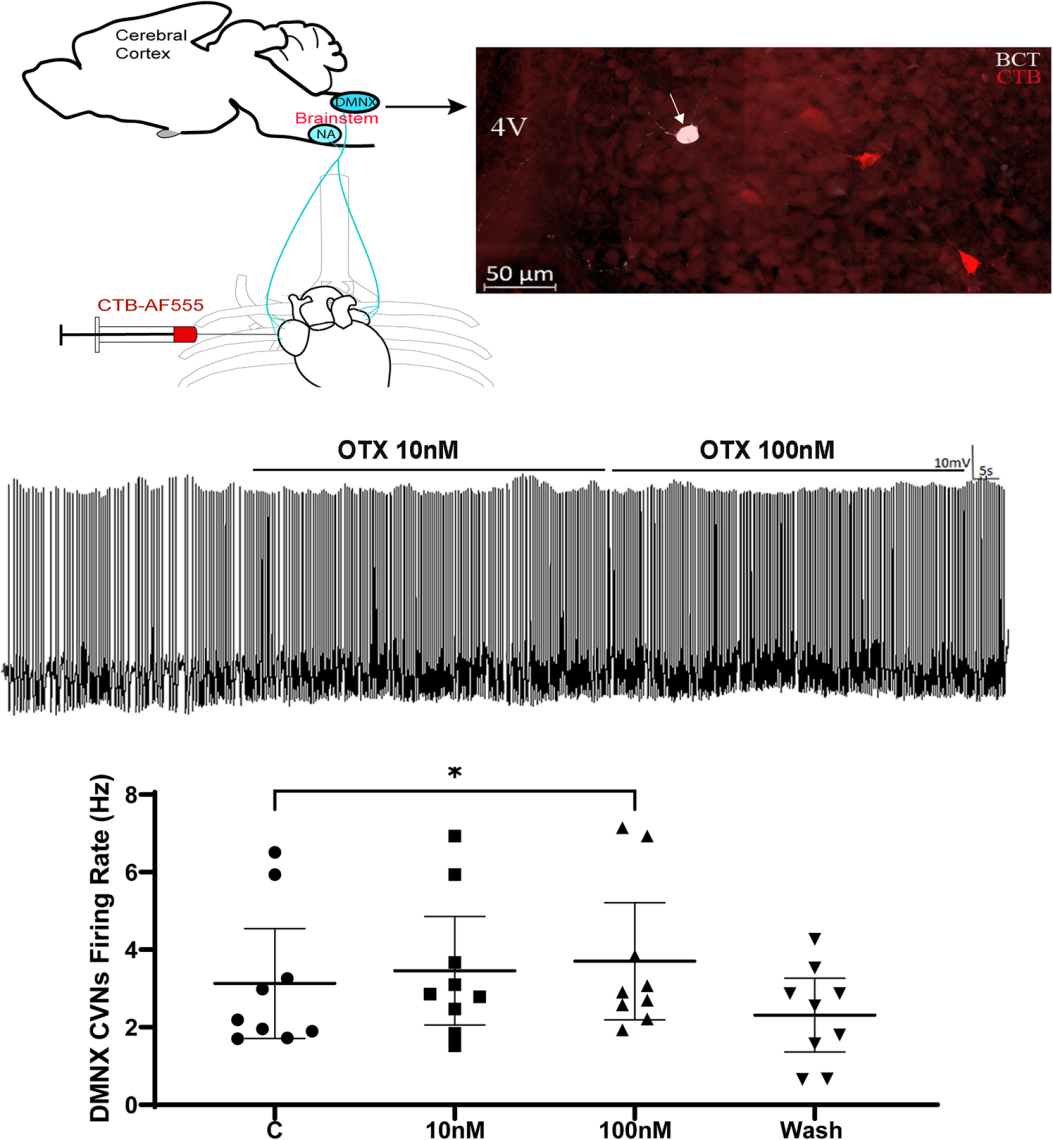
Administration of OXT enhanced CVNs action potential in the brainstem DMNX containing slice. Top left, Diagram illustrating procedure of retrograde labeling CVNs in the brainstem; top right, an example confocal image presenting CTB-555-labeled CVNS (red) and patched CVN neuron (red cell filled with biocytin in white); middle, typical spike trace showing OXT increased CVN neuron action potential firing rate; bottom bar graph presenting original data with 95% CI (*n* = 9 neurons from 7 animals). Data analyzed using RM one-way ANOVA with Dunnett's post hoc. *p < 0.05.

To test the effect of OXT on OXTR+ CVNs activity, additional studies examined their electrophysiological responses in CVNs in both NA and DMNX nuclei from seven crossbred OXTR-Cre × ChR2-eGFP animals (3 males and 4 females). Whole-cell patch-clamp electrophysiology recordings under current-clamp configuration were used to obtain and quantify spontaneous action potential firing rates before and after OXT administration. Application of OXT (100 nM) significantly increased the firing rate of CVNs in the DMNX by 20%, from 3.1 ± 0.6 Hz in control to 3.7 ± 0.6 Hz (*n* = 9 neurons from 7 animals, RM one-way ANOVA with Dunnett's multiple comparison, *F*_(1.352,10.82)_ = 6.147, *p* = 0.0241; Fig. 5). OXT had no significant effect on CVNs in the NA (data not shown).

### Chemogenetic activation of DMNX OXTR+ CVNs increases parasympathetic activity to the heart

**Figure 6.**
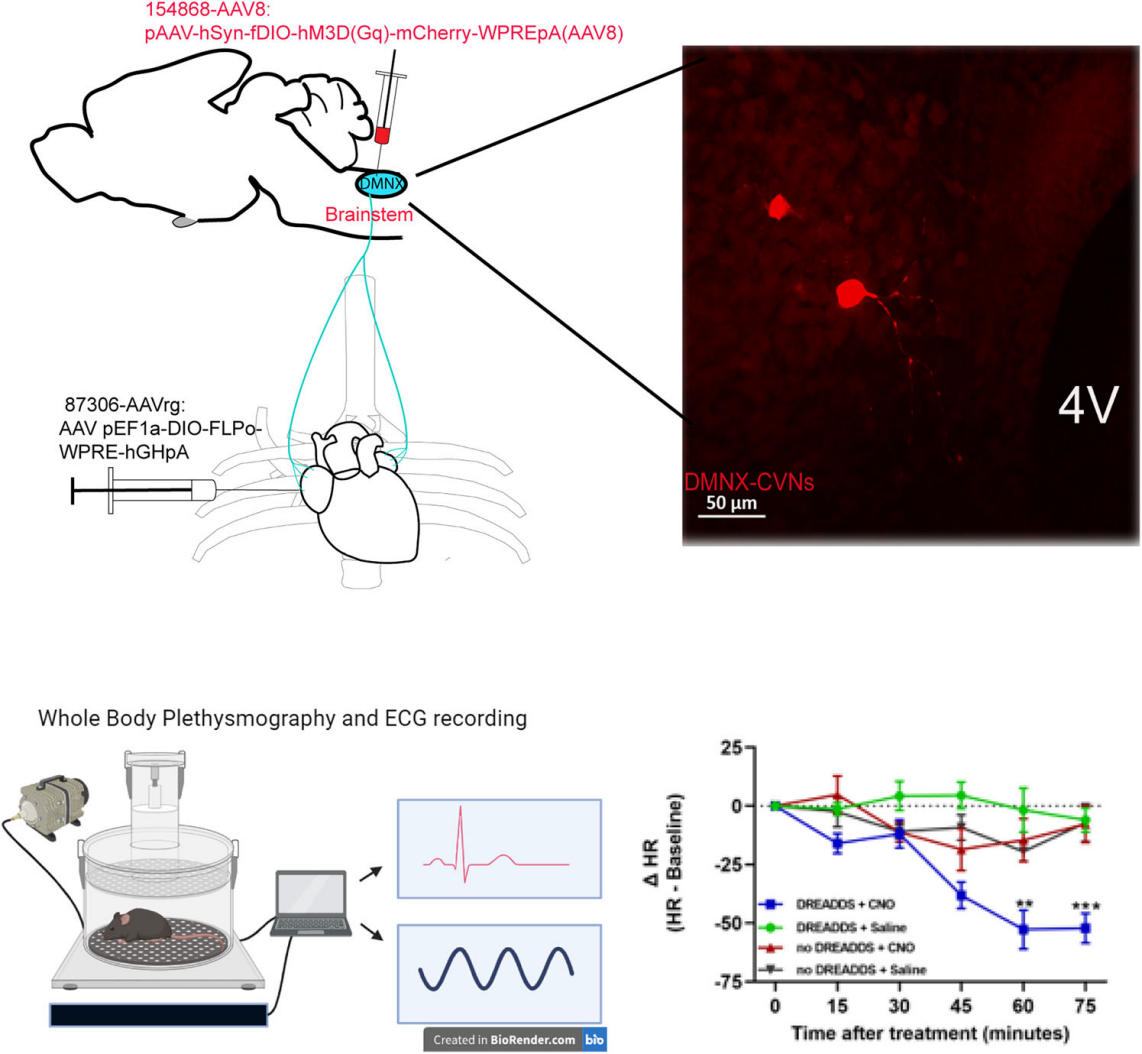
Chemogenetic activation of OXTR+ CVNs increases parasympathetic activity to the heart. Upper left diagram illustrating procedure of two-step viral vector injection to specifically identify and activate CVNs that project to the heart; top right, an example confocal image showing cells with mCherry expression in the brainstem slice (red); bottom left diagram illustrating plethysmography and ECG recording and bottom right presenting original data and statistic results. Two-way repeated-measures ANOVA with Tukey's post hoc **p < 0.001, ***p < 0.0001.

To determine whether excitation of OXTR+ CVNs in the DMNX could alter HR, DREADDs were expressed in OXTR+ CVNs using a retrograde Cre-dependent site-specific recombinase FlpO virus (Addgene AAV 87306) injected into the pericardial sac of male and female crossbred OXTR-Cre × ChR2-eGFP animals (*n* = 7, four males and three females), followed 4 weeks later with an injection of a Flp-dependent DREADD virus [Addgene AAV 154868-pAAV-hSyn-fDIO-hM3D(Gq)-mCherry] into the DMNX. Heart rate and respiratory functions were recorded with ECG and whole-body plethysmography. Administration of the DREADD agonist CNO (1 mg/kg, i.p. injection) in mice evoked a rapid and long-lasting bradycardia (Fig. 6), with a maximum HR reduction of 53 ± 8 bpm observed 1 h after CNO injection compared with baseline (60 min: *p* = 0.0074; 75 min: *p* = 0.0006; RM two-way ANOVA with Tukey's post hoc). There were no significant changes in respiration rate in any group. There were no significant changes in HR with CNO in non-DREADDs expressing animals or with saline injections in DREADDs expressing animals (*n* = 7 in each group).

## Discussion

There are four major findings from this study. Over half of the CVNs in the DMNX colocalize with OXT receptor positive neurons. Surprisingly, CVNs in the NA, as well as other ChAT neurons in the NA, have sparse expression of OXT receptors. OXT increased the firing of CVNs in the DMNX but had no effect in CVNs in the NA. Selective chemogenetic activation of OXTR+ CVNs in the DMNX elicited a rapid and long-lasting decrease in HR.

A long-standing issue in the field is whether the different populations of CVNs in the NA and DMNX play overlapping or different physiological roles. Anatomical work has shown CVNs originating from both the NA and DMNX project to cardiac ganglia (Sturrock, 1990; Standish et al., 1995) where their axons form extensive basket endings around ganglionic principal neurons (Cheng et al., 1999). However, other studies indicate neurons in the NA and DMNX innervate the same cardiac ganglia but target different subpopulations of principal neurons within the ganglia (Cheng et al., 2004). More specifically, fibers from the DMNX were found to project to small intensely fluorescent cells (possibly interneurons) as well as to cardiac principal neurons, whereas axons from NA neurons innervate only principal neurons. Lesions of the NA almost completely abolished the baroreflex control of the HR, whereas lesions of the DMNX did not, suggesting that the NA plays a more important role in baroreflex control of HR than the DMNX (Cheng et al., 1999). Other work found the majority of DMNX CVNs have no obvious input from central or peripheral respiratory or cardiac-related inputs but are activated by stimulation of pulmonary C fiber afferent fibers, suggesting DMNX CVNs may play a more important role in pulmonary C-fiber-evoked bradycardia (Jones et al., 1998). In addition, NA CVNs are primarily acetylcholinergic while DMDX CVNs release acetylcholine and neuropeptides as cotransmitters (Coverdell et al., 2022). It is also worth noting that, using chemogenetic activation, long-term stimulation of only CVNs in the DMNX reduced HR (Strain et al., 2024) and blood pressure in the spontaneously hypertensive rat (Moreira et al., 2018).

OXT is only synthesized in a limited number of discrete brain regions: the PVN and supraoptic and accessory nuclei of the hypothalamus (Son et al., 2022). While many brain areas receive dense projections from OXT neurons, and a multitude of neuronal populations strongly express OXT receptors, there is surprisingly little spatial correlation between OXT fibers and receptors. Only a few neuronal populations both receive dense OXT fibers and have robust expression of OXT receptors. The medulla has the highest positive correlation ratio of OXT fibers to OXT receptors in the CNS (Son et al., 2022). Prior work from our lab, and others, have shown there are dense projections from PVN OXT neurons to nuclei in the medulla, and more specifically, there is a monosynaptic excitatory pathway from PVN OXT neurons to CVNs in the DMNX and that activation of the fibers in this pathway releases OXT (Pinol et al., 2012, 2014; Jameson et al., 2016; Garrott et al., 2017; Dyavanapalli et al., 2020b; Rodriguez et al., 2023; Schunke et al., 2023). Neurons in the DMNX have previously been shown to have a robust expression of OXT receptors (Son et al., 2022). OXT has been shown to excite rat DMNX neurons in vivo in prior work using extracellular (Charpak et al., 1984) and intracellular recordings (Raggenbass et al., 1988) as well as in in vitro slices (Dubois-Dauphin et al., 1992), although the excitation of DMNX neurons with OXT was absent in DMNX neurons from the guinea pig (Raggenbass et al., 1987). Microinjection of OXT into the NTS-DMNX complex has been shown to potentiate the bradycardic responses to pressor challenges, which was absent after blocking parasympathetic activity with atropine (Higa et al., 2002). The results in this study advance this work by demonstrating a majority of CVNs in the DMNX are among only a few neuronal populations that receive dense OXT fibers that release OXT and have robust expression of postsynaptic OXT receptors. OXT increases the firing of OXTR+ CVNs in the DMNX, and chemogenetic activation of OXTR+ CVNs decreases HR. Future work is needed to test if selective activation of OXTR+ CVNs in the DMNX prevents or treats autonomic imbalance and dysfunction in cardiovascular and other diseases (Wang et al., 2019; Jankowski et al., 2020). Additionally, as a unique population of OXTR+ CVNs in the DMNX has been identified that when stimulated decreases HR, additional work is needed to identify coactivators of this specific subpopulation of OXTR+ CVNs.

## Synthesis

Reviewing Editor: Charlotte Cornil, Universite de Liege Decisions are customarily a result of the Reviewing Editor and the peer reviewers coming together and discussing their recommendations until a consensus is reached. When revisions are invited, a fact-based synthesis statement explaining their decision and outlining what is needed to prepare a revision will be listed below. The following reviewer(s) agreed to reveal their identity: Kirsteen Browning.

This manuscript has now been reviewed by two experts who both agree that the methods and results are technically solid, although important questions were raised that need to be addressed. Whether the results are genuinely novel, beyond confirming with modern tools what was previously known, was debated and we feel that the novelty of this work is not sufficiently clear. The authors are thus invited to make their case more convincing.

Reviewer comments

Review editor:

Please provide information about the sex of the animals used in each experiment.

Reviewer 1

The role of cardiac vagal neurons (particularly within the DMNX) is grossly understudied relative to other vagal neurons as well as cardiac sympathetic neurons. The clinical efficacy of oxytocin in modulating parasympathetic activity has interesting cross-disciplinary translational relevance and the mechanistic basis of these actions should be explored in more depth.

The authors have presented solid evidence that OXT receptors (OXTR) are differentially expressed in cardiac vagal neurons with the DMV but not NA, and that chemogenetic excitation of these OXTR expressing neurons induced bradycardia.

The experiments have been conducted in a technically appropriate manner and the conclusions drawn from the results appear appropriate. Nevertheless, I have some questions/comments for the authors:

What was the rationale behind retrogradely labeling preganglionic parasympathetic cardiac vagal neurons at P5 and then performing experiments 3-10 or so days late? The descending hypothalamic oxytocin innervation is not fully patterned until quite later during postnatal development, and oxytocin receptor distribution shows a similar long postnatal maturation. Is it not possible that patch clamp recordings and confocal imaging were made from immature neurocircuits, the developmental maturation of which was different from those mice in which vivo heart rate measurements which occurred much later after chemogenetic transfection?

Given the potential for metabolism of CNO to clozapine, why were clozapine control experiments not conducted, particularly when measuring heart rate responses?

The change in DMNX CVN excitability as recorded by action potential firing rate was relatively modest (3.1Hz to 3.7Hz, or approximately 20% increase), yet the bradycardia was much more profound. Any explanation for the discrepancy?

Reviewer 2

The present study examined the role of oxytocin on controlling cardiac autonomic regulation of the heart when acting in either the dorsal motor nucleus of the vagus (DMNX) or the nucleus ambiguous (NA). This work appears to have been well-done, and I share the authors' enthusiasm for further research on the cardioprotective effects of oxytocin. The paper describes its major findings as 1) Most DMNX cardiovascular neurons express oxytocin receptors, 2) cardiovascular neurons in the NA do not express oxytocin receptors, 3) oxytocin increased the firing of cardiovascular neurons in the DMNX and had no effect in the NA, and 4) activating oxytocin receptor-expressing cardiovascular neurons in the DMNX slowed heart rate.

However, I fear this work suffers from a common phenomenon in neuroscience research of revisiting well-established effects with newer methods and claiming novelty. When the abstract concludes "DMNX CVNs expressing OXTR with oxytocin may represent a novel translational therapeutic target", I was reminded of the work of Charpak et al., which established the excitatory effect of oxytocin on neurons in the dorsal motor nucleus in 1984. A few years later, that same lab established the presence of oxytocin receptors in the DMNX via autoradiography. I am unaware of any shortcomings in autoradiography or the combination of pharmacology and electrophysiology that would warrant a return to this. The other major finding of this work, that nucleus ambiguus lacks oxytocin receptors is in accord with the findings of the Mendelowitz lab's 2002 study of neuropeptide actions in that nucleus, which found that oxytocin did not affect neural activity there. The 2002 work of Michelini's lab, "Baroreflex control of heart rate by oxytocin in the solitary-vagal complex" is also relevant, having showed that oxytocin administration into the DMNX slows heart rate. Unfortunately, this leaves the present manuscript with not having clearly demonstrated any novel contributions to the field.

Specific comments:

ABSTRACT

When the abstract states "Recent studies have identified...", it strains the definition of 'recent', since these functional anatomical connections have been known for decades. Similarly, the Introduction repeats this description of 'recent' and then cites papers from 2012 and 1996. While my knees may wish that 2012 was recent, it is not.

METHODS

Line 73 - "Animal Care and Use Guide- lines, approved by the [Author University] Institutional Animal Care and" needs to be completed.

DISCUSSION

Line 302, "Future work is needed to test if selective activation of OXTR+ CVNs in the DMNX prevents or reverses autonomic imbalance and dysfunction in cardiovascular and other diseases" could instead refer to any of several reviews on this topic, e.g. Jankowski 2020 in Frontiers in Psychology, or Wang et al. 2019 in Frontiers in Neuroscience.
